# Molecular complexity analysis of the diagnosis of Gitelman syndrome in China

**DOI:** 10.1515/biol-2022-0634

**Published:** 2023-06-20

**Authors:** Wei Song, Yue Hu, Ling Zhao, Jinming Zhang, Yu Zhang, Jianxuan Wen

**Affiliations:** The Second Affiliated Hospital of Guangzhou University of Chinese Medicine, Guangzhou, Guangdong Province, 510120, China; Second Clinical College of Guangzhou University of Chinese Medicine, Guangzhou, Guangdong Province, 510120, China; Department of Endocrinology, Guangdong Provincial Hospital of Chinese Medicine, No. 55 Inner Ring West Road, University Town, Panyu District, Guangzhou 510120, China

**Keywords:** Gitelman syndrome, hypokalemia, SLC12A3

## Abstract

Gitelman syndrome (GS) is an autosomal recessive renal tubal disease characterized by hypomagnesemia, hypokalemia, and hypocalciuria. The disease is caused by defects in the SLC12A3 gene, which encodes the thiazide diuretic-sensitive sodium chloride cotransporter (NCCT). In this study, a 20-year-old female patient with recurrent hypokalemia was tested for a hypokalemia-related panel using Next Generation Sequencing. Pedigree analysis was performed on her parents (non-consanguineous) and sister using Sanger sequencing. The results revealed that the patient carried compound heterozygous variants of the SLC12A3 gene: c.179C > T (p.T60M) and c.1001G > A (p.R334Q). Furthermore, her asymptomatic 6-year-old sister also carried both mutations. While the p.T60M mutation had been reported previously, the p.R334Q mutation was novel, and amino acid position 334 was identified as a mutation hotspot. Our findings provide an accurate molecular diagnosis that is essential for the diagnosis, counseling, and management of not only the symptomatic patient but also her asymptomatic sister. This study contributes to our understanding of the GS, which has a prevalence of approximately 1 in 40,000 and a heterozygous mutation carrier rate of 1% in Caucasians. Specifically, we observed a compound heterozygous mutation of the SLC12A3 gene in a 20-year-old female patient presenting with clinical symptoms consistent with GS.

## 1 Introduction

Gitelman syndrome (GS) is also called familial hypokalemia and hypomagnesemia. The characteristic clinical features are hypokalemia, hypomagnesemia, hypocalciuria, metabolic alkalosis, and low or normal blood pressure, which was first described by Gitelman in 1966 [1]. It is an autosomal recessive renal tubal disease with a prevalence of about 1 in 40,000 [2], and the heterozygous mutation carrier rate in Caucasians is 1% [3]. It is the most common salt-loss renal tubular genetics disease [4]. Most patients are onset at youngster or adult stage, but some can also occur during childhood. In addition to fatigue, weakness, and other clinical manifestations of low-potassium, other symptoms are mild, or unrecognized. A small number of patients may have low-magnesium symptoms like facial numbness and seizures, especially onset after fever, vomiting, and diarrhea.

GS is caused by mutation in the SLC12A3 gene encoding the thiazide diuretic-sensitive sodium chloride cotransporter (NCCT) [5]. The SLC12A3 gene contains 26 exons and encodes 1,030 amino acids. It encodes the distal tubular thiazide diuretic sensitive ion channel NCCT [6]. The NCCT protein is comprised of various regions, including the transmembrane region, intracellular carboxyl terminus, and extracellular hydrophobic ring region. It is primarily expressed in the parietal membrane of the lumen epithelial cells of the distal convoluted tubule (DCT), which is responsible for reabsorbing approximately 7% of the total sodium chloride filtered through the glomeruli. When NCCT dysfunction occurs, the DCT is unable to effectively reabsorb sodium, leading to increased urinary sodium excretion, decreased blood volume, and subsequently decreased blood pressure. Moreover, this dysfunction can activate the renin-angiotensin system, resulting in a reduction in potassium and metabolic alkalosis [7]. The decrease in urinary calcium may be related to the decrease in intracellular hyperpolarization and the increase in resorption due to abnormal combined transport. Hypomagnesemia is one of the characteristics of GS which is different from Batter Syndrome, but its mechanism is still unclear [3]. In GS patients, the mutated gene may be downregulated urinary magnesium production, leading to hypomagnesemia [8]. In addition, the role of aldosterone in the formation of lumen side negative potential may also contribute to increased urinary magnesium and decreased blood magnesium, as it increases Na^+^/Mg^2+^ exchange. Despite more than 400 reported mutations in the SLC12A3 gene, only one mutant allele is typically identified after SLC12A3 screening in 18–40% of patients presenting with clinical symptoms of GS.

## 2 Case Presentation

### 2.1 Medical history

The patient is a 20-year-old female who has been experiencing recurrent limb weakness, paroxysmal paralysis, and hypokalemia since the age of 16. Her blood potassium levels were found to be low, but symptoms resolved after potassium supplementation. At 18, she was hospitalized due to limb weakness, and again low blood potassium levels were identified, but her blood pressure, thyroid function, renin-angiotensin-aldosterone, and adrenal CT enhanced scan were all normal. She was prescribed potassium chloride tablets, which effectively treated her symptoms, and experienced recurrent episodes of the same symptoms which were again resolved with potassium supplementation.

Six days before admission, the patient once again exhibited limb weakness and spasms, accompanied by palpitations, with a measured blood potassium level of 2.15 mmol/L. The electrocardiogram showed sinus tachycardia and normal blood pressure. She denied experiencing nausea, vomiting, recurrent abdominal pain, diarrhea, polyuria, polydipsia, and had no history of taking diuretics or other drugs. Her diet, sleep, and development were normal, and there was no family history of similar diseases. Both her parents and her 6-year-old sister were healthy.

The patient’s physical examination revealed no abnormalities in her heart, lungs, or abdomen, and the Trousseau sign and Chvostek sign were negative. Blood tests showed that her potassium level was 2.46–3.39 mmol/L, magnesium level was 0.32–0.37 mmol/L, chlorinate level was 141 mmol/L, chlorine level was 97.6 mmol/L, calcium level was 2.47 mmol/L, and phosphorus level was 1.260 mmol/L. Additionally, we performed a 24 h urine test to measure potassium, magnesium, chlorine, sodium, and calcium levels, which indicated renal potassium loss. Sex hormone results were as follows: TSTO 1.79 nmol/L; FSH, LH, PRG, and E2 were within normal limits. Blood gas analysis showed a pH of 7.581, PaO_2_ of 113 mmHg, PaCO_2_ of 27.3 mmHg, HCO_3−_ of 26.6 mmol/L, and a base excess of 4.7 mmol/L. Thyroid function, blood cortisol, and adrenocorticotropic hormone, liver and kidney function, renin-angiotensinaldosterone function, adrenal enhancement CT, abdominal color Doppler ultrasonography, urinary color Doppler ultrasonography, and electrocardiogram were all normal.


**Informed consent:** Informed consent has been obtained from all individuals included in this study.
**Ethical approval:** The research related to human use has been complied with all the relevant national regulations, institutional policies, and in accordance with the tenets of the Helsinki Declaration, and has been approved by the Ethics Committee of Guangdong Provincial Hospital of Chinese Medicine.

### 2.2 Clinical material collection

During hospitalization in our department, the patient underwent a series of tests, including blood tests such as blood routine, blood potassium, blood sodium, blood chlorine, blood magnesium, blood calcium, blood phosphorus, liver and kidney function, heart enzyme levels, blood gas analysis, blood cortisol and adrenal cortical hormone rhythms, sex hormones, growth hormone, and high blood pressure (aldosterone, renin activity, angiotensin); urine tests such as 24 h urine (potassium, sodium, chlorine, magnesium, calcium, and phosphorus) and urine routine; and other examinations such as ECG, DR chest radiograph, and kidney + adrenal CT scan + enhancement.

### 2.3 Target capture and sequencing

2–5 mL blood samples from the patient were collected in EDTA-coated tubes. DNA was extracted using SolPure Blood DNA kit (Magen). Hypokalemia panel was performed using next generation sequencing (NGS). Briefly, DNA was fragmented by Q800R Sonicator (Qsonica) to 300–500 bp size fragments. The libraries were prepared according to Illumina library preparation protocol. Target genes were captured by custom designed NimbleGen SeqCap probes (Roche NimbleGen, Madison, Wisconsin). DNA samples were sequenced by NextSeq500 (Illumina, San Diego, California).

### 2.4 Bioinformatics analysis

In this study, sequencing data in FASTQ format underwent filtering to obtain “clean reads” which were then mapped to the human genome 19 (2009-02 release, http://genome.ucsc.edu/). Nucleotide changes were identified and reviewed with NextGENe 2.4.1.2 (SoftGenetics, State College, PA), while copy number variants were detected using eCNVscan software. Population databases such as 1000 Genomes, dbSNP, and GnomAD were used to annotate the variants, and previous articles were scrutinized with the Human Gene Mutation Database, Leiden Open Variation Database, ClinVar, and Google Scholar.

Computational prediction of variants was carried out using PolyPhen-2, SIFT, and MutationTaster, with interpretation of the variants being guided by the American College of Medical Genetics and Genomics (ACMG) guidelines.

### 2.5 Sanger sequencing

To verify the genetic variation identified in the patients’ relatives, Sanger sequencing was conducted. Primers were designed and synthesized specifically for the mutation, and genomic DNA was extracted from the blood samples. A specific PCR reaction system was used to amplify the targeted sequence containing the mutation, and the resulting PCR product was purified prior to Sanger sequencing. The peak results of the sequencing data were analyzed using the Mutation Surveyor analysis software.

### 2.6 Bioinformatics analysis

To analyze the protein structure and predict conservation and functional domains, we employed mutation prediction software such as PolyPhen-2 (http://genetics.bwh.harvard.edu/pph2/) and SIFT (http://sift.jcvi.org/www/SIFT_BLink_submit.html). Multiple sequence alignment was performed as well. Evolutionary conservation across species was assessed through CLUSTAL analysis (http://www.ebi.ac.uk/Tools/msa/clustal). Additionally, we used the self-optimized prediction method with alignment (SOPMA, https://prabi.ibcp.fr) to predict the secondary structure.

## 3 Results

### 3.1 Genetic testing results

**Figure 1:**
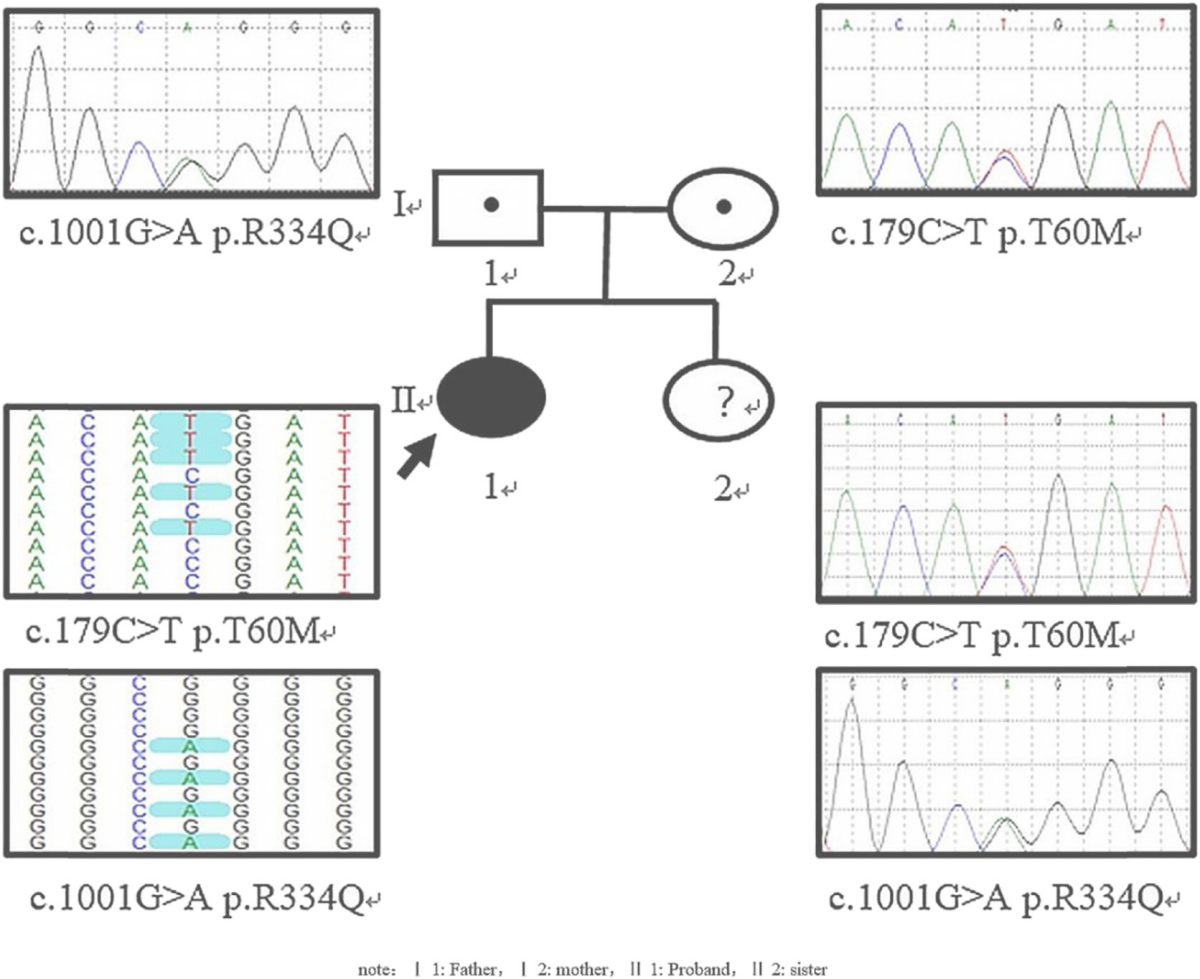
Genetic testing results of patient’s pedigree.

NGS revealed two heterozygous variants of SLC12A3 gene, c.179C > T (p.T60M) and c.1001G > A (p.R334Q). Sanger sequencing showed that the variant c.179C > T (p.T60M) was inherited from the mother and c.1001G > A (p.R334Q) was inherited from the father. Therefore, the two variants constituted a compound heterozygous state. In addition, Sanger sequencing revealed that the patient’s 6-year-old sister also carried the same compound heterozygous mutations, but she did not show clinical manifestations since most GS patients were onset in youngster or adult stage only (Figure 1).

### 3.2 Bioinformatics analysis results

**Figure 2:**
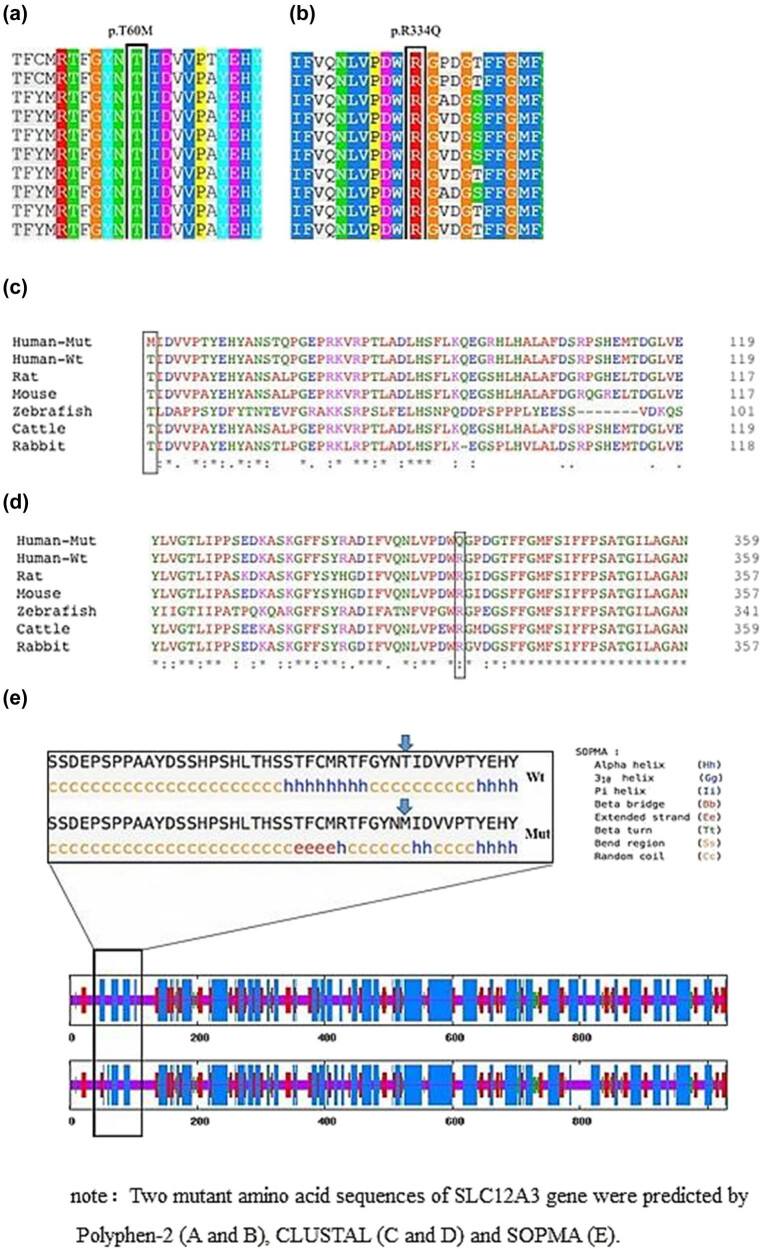
Bioinformatics analysis of SLC12A3 gene variation. Two mutant acid sequences of SLC12A3 gene were predicted by PolyPhen-2 (a) and (b), CLUSTAL (c) and (d), and SOPMA (e).

Polyphen-2 predicted both p.T60M and p.R334Q variants to be probably damaging, and these mutations were found to be highly conserved during evolution (Figure 2a and b). CLUSTAL analysis confirmed that Thr residue at position 60 and Arg residue at position 334 of the SLC12A3 protein were highly conserved, indicating their crucial biological function (Figure 2c and d). The substitution of Thr and Arg residues by Met and Gln, respectively, altered the physiochemical properties of the conserved amino acid residues, which in turn could affect the protein’s spiral structure and overall function.

Furthermore, secondary structure prediction by SOPMA demonstrated that substitution of Thr at residue 60 led to significant alteration of the protein secondary structure (Figure 2e). This structural perturbation was likely to impair protein function or cause damage. Conversely, the substitution of Arg at residue 334 did not significantly alter the protein’s secondary structure.

## 4 Discussion

Glomerulonephritis with associated hypokalemia-hypomagnesemia syndrome (GS) presents with significant individual variation in phenotype. It has been suggested that serum magnesium levels could impact phenotype. Research shows that male patients often display more symptoms than females, which may be linked to hormone levels. Even with the same mutation, female patients can present differently from their male counterparts. Further studies are warranted to explore the relationship between genotype and phenotype.

Management of GS commonly involves a high-sodium, high-potassium diet supplemented with potassium chloride and magnesium preparations. Magnesium aspartate, magnesium lactate, and magnesium chloride are deemed to be more bioavailable when compared to magnesium sulfate and magnesium oxide. However, it is important to note that oral administration of large amounts of magnesium preparations could lead to diarrhea. Typically, magnesium chloride is recommended at a dosage of 4–5 mg/(kg/day), administered in 3–4 doses to prevent diarrhea. Additionally, aldosterone antagonist spironolactone or collector epithelial sodium channel (ENaC) inhibitor (amiloride) should be used concurrently to reduce urinary potassium excretion.

The Human Gene Mutation Database shows that until 2014, 425 mutations had been reported in the SLC12A3 gene, with missense mutations being the most common, and heterozygous mutations being more common than homozygous mutations. Most patients present with two different mutation sites. The p.T60M mutation is the most prevalent amino acid mutation site in the Chinese and Asian populations [9,10], while IVS9 + 1G > T (c.1180 + 1G > T) is the most common mutation among Europeans [11]. The mechanisms by which NCCT function is impaired or lost due to mutation may include reduced protein synthesis, weakened binding of functional proteins to membranes, weakened function of synergistic transporters, and accelerated protein transport or degradation.

These two rare missense variants were identified in this patient. The p.T60M variant has been reported in clinical cases of hypokalemia, while the p.R334Q mutation has not been reported in clinical cases, yet its mutation region is a hotspot in this gene. Other pathogenic mutations in the same amino acid location, p.R334W and p.R334P, have been reported in clinical cases. The allele frequencies of these two mutations in global and East Asian populations are low in the GnomAD population database. Furthermore, the amino acid sequences of these mutations are highly conserved among different species (Figure 2a and b). According to ACMG mutation classification guidelines, these two mutations are classified as “likely pathogenic.”

The patient’s sister also carries these two pathogenic mutations, p.R334W and p.R334P, in the SLC12A3 gene, but she has yet to display any clinical phenotype of GS, given that GS is an adult-onset disease. As her sister is only 6 years old, regular clinical inspections of blood potassium and magnesium levels are recommended to enable early prevention and treatment by physicians.

In summary, identifying this novel SLC12A3 gene mutation expands the mutation spectrum and underscores the genetic heterogeneity of GS. This finding highlights the importance of molecular genetic diagnosis for individuals with GS carriers. The genetic testing results of this patient and her relatives provide additional support for phenotypic-genotype analysis of GS, which could facilitate early diagnosis, genetic counseling, and management of GS.
